# Memoranda of Cases of Cerebro-Spinal Meningitis

**Published:** 1872-06

**Authors:** W. C. Lyman


A MONTHLY RECORD OF

*Medicine, Surgery and the Collateral Sciences.*

EDITED BY J. ADAMS ALLEN, M.D., LL.D.; AND WALTER HAY, M.D.

VOL. XXIX. — JUNE, 1872. — NO. 6.

### Original Communications.

ARTICLE I.—*Memoranda of Cases of Cerebro-Spinal Meningitis.* Read before the Chicago Society of Physicians and Surgeons, by W. C. LYMAN, M.D.

PRACTICE OF DR. FISHER.

Case 1. Infant, 7 weeks old. Family in comfortable circumstances, and commodious, well-ventilated rooms. Seized with alarming symptoms after apparently excellent health. Dead in 20 hours. Petechial eruption, almost purpura, universal; opisthotonos; heavily loaded tongue; no vomiting nor diarrhœa. Bromid. potass., and, at first, alterative cathartic.

Case 2. Young man, 16 years old. No eruption; intense cervical pain and slight opisthotonos; disturbed secretions. Recovery in two weeks, after use of Bromid. pot., grs. x, Tr. cannab. ind. gtts. xx at dose.

DR. HYDE.

Case 1. Adult, 35 years old. No eruption; intense cephalalgia and cervical pain, deep seated; frequent small pulse; tongue not loaded; eyes sunken and surrounded by dark rings. Duration two weeks. Bromide quinia; blisters, and, finally, beef tea.

Case 2. Infant, 5 weeks old. Loaded tongue; diarrhœa, vomiting; hot, pungent skin; rapid pulse; opisthotonos; eruption viscid, and disappearing in 48 hours; sunken eyes; full fontanelle; stupidly quiet when not aroused, then fixed stare with eyeballs turned upward. Improvement under alterative cathartic; counter-irritation of spine; lime water and milk; warm baths, and quinia afterward. Relapse in two weeks; symptoms similar, but not so severe. Quinia and counter-irritation of spine, with baths. Recovery.

Case 3. Infant, 7 weeks old. High fever; great stiffness of neck, especially in upright position; evidently great pain on motion; vomiting moderate; dejections discolored; eruption dark and apparently confined to back and shoulders; stupid when left alone, and reclining with head thrown as far back as possible; fontanelle not noticeably changed; spine tender, and covered with eruption in cervical portion; emaciation rapid; eyes sunken; tongue partly white. Treatment similar to preceding, including bromide. Two days after, manifest improvement. Moderate relapse. Recovery.

DR. EMMONS.

Case 1. Minnie W.; age 4½ years. Douglas Place; called April 23. High fever; great capillary congestion; dark, mottled spots on body; pulse 150; resp. 28 or 30. Pres. hyd. and chlorate potassa. Called at noon; no abatement of fever. Prescribed belladonna and bromide; cold applications to head; counter-irritation to spine. Called in the evening. Applied blister to spine; gave quinine in large sedative doses, and continued bellad. and bromide. Patient continued to sink, and died at 11 o'clock. Duration of disease, 26 hours.

Case 2. Girl; age 8 mo.; in Bridgeport. Called May 19. Frequent vomiting; high fever; erythematous eruption of legs and body; eyes sunken, with dark circles; constipated; tenderness of spine and neck. Gave bromide and belladonna, with hyd. subchlor. to move bowels. In 12 hours eruption subsided, followed by capillary congestion; no abatement of fever; still vomiting. Applied leeches to spine. Capillary congestion immediately subsided; troublesome hæmorrhage from bites. No return of congestion since leeching; some improvement. Gave quin. and neutral mixture and ferri elix. phos. Is still under treatment.

*Later* (Sunday). Progressed favorably up to date. Found more feverishness, culminating in a vesicular eruption, after which fever subsided. Continues to improve. Continue treatment as before.

DR. WILDER.

Case 1. —— Hawley; girl, 7 years old. Called noon, March 26, 1872. Had congestive chill and convulsions; tongue coated, and fever; rigid neck; vomiting bile. R. Dil. hydrocyanic acid and tinct. calabar bean, with alterative, followed by sulph. quin.; warm bath, and mustard on neck and over stomach. At 5 p. m. two more convulsions; strabismus marked; neck more rigid; pain in back; pulse about 90. Dr. Dyas was called in counsel. Continued treatment: hyd. mit. chlo. grs. x, followed by oil; six leeches back of ears and blister along spine; iodide and bromide of soda, with tinct. calabar bean. Became convalescent in eight days; remained prostrate four weeks. Hygienic surroundings good. (Cor. Butler and Kossuth streets.)

Case 2. Miles Kearney, 170 21st street. Age, 1 year. Called April 4. Had been sick one week. A little strabismus; neck slightly rigid; no convulsions; pneumonia as secondary complication. Treated with bromides and calabar bean; mild counter-irritation on chest. Recovery in three weeks.

Case 3. Grace Egan, 22 Blackwell street. Age 5 years. Sick two days from April 10. Opisthotonos; vomiting; convulsions; pulse very rapid and weak; rapidly subsided into unconsciousness. Symptoms nothing special except the rapid decline. Treatment same as preceding cases. Death in 48 hours.

DR. TRIMBLE.

Case 1. A girl two years old, residing on Johnson street, West Div. Dirty locality, though the house kept in a neat and clean condition. Patient had been ill for six weeks when I first visited her on the 5th of the month (May). Symptoms: Fever; nausea, and very frequent vomiting; constipation; great irritability of temper; anorexia; pupils somewhat dilated; surface pale. Treatment: Two doses of castor oil were given in the first three days; a diaphoretic mixture (citric acid, carb. potass., spts. nitri dulc.) brom. potass., elix. calisaya and pyrophosph. iron; stimulating lotion to spine, (spts. ammon., chloroform, alcohol, glycerine); chicken tea; farinaceous preparations and gelatine as nourishment.

May 20. Has had no fever, and vomited but twice during the last two days. Continued the tonic; ordered a little Madeira wine twice a day, and diminished the brom. potass. 26th. Vomiting increased; also spinal tenderness; serous effusion undoubted; result uncertain.

Case 2. Boy, 3½ years old, residing on De Puyster street, West Div.; a very clean little street, paved; house comfortable and in good order. Visited him first on 13th May. Eight weeks before that date, a sister, 8 years of age, died, and the same day this patient was attacked. He had been attended at first by a West Side physician, and afterwards by Prof. Davis, who left him in my care during his absence at the National Medical Convention. Symptoms: High fever; pupils greatly dilated; frequent jerking motion of the legs, especially the left; toes drawn downwards, but readily relaxed by friction; tremulous motion of the hands, especially the right hand; loss of speech, though the mind was clear; constipation; some vomiting; pallid countenance, sometimes of dusky hue about the mouth and eyes. On the 17th, pale red spots, about half an inch in diameter appeared on the face, but had disappeared at next visit, on the 20th. At my first visit, he had not spoken for two weeks. On the 20th spoke without difficulty; fever much diminished; vomiting ceased; bowels regular; motion of the hands and feet ceased; patient decidedly better. Treatment: brom. potass., to which I subsequently added the iodide; the same tonic; diaphoretic, and local stimulant, as in Case 1. 26th. Speech again lost; partial paralysis of right side; pupils dilated; effusion probable. Result doubtful.

Case 3. An office patient; a boy 2½ years old; had been sick for several weeks, but had recovered except the loss of hearing and inability to stand, on account of weakness of the back. He could use his limbs actively, while in the recumbent or sitting position.

I have had several patients who have exhibited some of the symptoms of the disease, as a tendency to opisthotonos, pains in the muscular system, fever, nausea, etc.

DR. MONTGOMERY.

Case 1. April 9. Infant, 7 months old. Intense congestion of the vessels of the head; pulse high; throbbing of carotids; great pain; cold hands and feet; pupils dilated; tongue dry; lip retracted; condition typhous. Gave bromide of soda and potass. 10th. Greater prostration; petechia. Mercurial purge; continue bromides. Patient unconscious; pupils dilated; muscles of neck rigid. 11th. Patient some better. Continue bromides, with 3-drop doses of Tr. calabar bean. 12th. Pulse 110; resp. 30. Child better. 13th. Discontinued cal. bean. Gave small doses of bromid. pot. May 3. Discharged; well.

DR. LYMAN.

Case 1. March 28. Child of 10 years; well grown girl. Suffered from lassitude the day before. Found patient comatose, with cold feet and hands; pale, with dilated pupils. Gave mercurial purge, with blister to nape of the neck, and sinapisms to feet. Free purging, followed with return of consciousness after 20 hours; then high fever of sthenic type; full pulse, about 120 per minute, and intense pain in head and spine. Ordered hot bath with bromide potass. and veratrum viride; cold to head, and purgative. Great relief followed. Next day a return of the fever, pain in head and racking pain in the spine of extraordinary severity. Repeated hot bath; gave morphia; large anodyne poultice to spine. Effect of opiate highly satisfactory. There was a daily accession of fever and pain for a week, over which the bromides, variously used, with belladonna, hyoscyamia, cannabis indica, aconite and veratrum viride, had no apparent control; the opiates only giving that relief from excessive pain without which the patient would have immediately succumbed, producing quiet sleep, composure of the mental faculties, and improved vital condition. At the end of the first week there was established a *habit* of pain in the afternoon, it coming on about one, and passing off at four o'clock. Quinine would not interrupt the paroxysm. Used to give an opiate to anticipate it. Urine pretty full in quantity throughout the disease. At the end of the second week the febrile symptoms became nearly intermittent, when quinine was given with good effect on the general condition but did not interrupt the afternoon fever. A considerable degree of headache remained after the fever had subsided, the mental faculties resumed their natural condition, and the tongue cleaned. This yielded to a succession of blisters behind the ears, and on temporal spaces. Has had no eruption. The only special points of this case: the sthenic type of the inflammation, extraordinary pain in the spine, and the great utility of opiates. Patient under treatment 35 days. Complete recovery.

Case 2. Child, 2 years old; in same house as the first. Ill with symptoms of ordinary bronchitis one day, and showed great dilatation of pupil, nervous twitching and tetanic convulsions by noon next day, from which the child died at 10 P. M.

Case 3. Child, 2½ years old, German parentage. Headache; fretfulness; slight dilatation of pupil; obstinate constipation. Treated by mercurial purge, sinapisms to spine, bromide potass. and tonics. Convalescent in ten days.

Case 4. Child, less than 2 years old; robust boy. Dilatation of pupil, opisthotonos; great photophobia; constipation; intense fretfulness. Purge; blister to cervix, followed by quinine immediately, as he had daily an almost complete remission of the febrile symptoms. This case had a troublesome cough, which yielded to bromide potass. Convalescent in two weeks.

Case 5. Child, 8 years old, living on 20th street. Ill April 28. Saw the child on the night of the 29th. Had had convulsions of tetanic character; was lying comatose; pulse 140, small and weak; face dusky; tongue dry; great throbbing of carotids; engorgement of the whole cerebral circulation; had all the appearance of typhus; obstinate constipation; respiration hurried, with occasional sigh. No special spinal symptoms. Gave mercurial cathartic, with blister to cervical portion of spine; hot foot baths; cold to head, and full doses of bromide potass. Next morning consciousness had returned; had darting pains in head, of extreme severity. Full doses of bromide, with hyoscyamia, and afterward chloral, only partially relieved it. Found it necessary to give opiates to procure any sleep or comfort. They were always followed by a more satisfactory condition of pulse and skin. Unlike Case 1st, this was wholly of asthenic type, the pulse frequent and feeble; face dusky; tongue dry; bowels obstinately constipated; temper peevish; periods of heightened fever, occurring irregularly during the day, with no regular exacerbation; urine constantly tolerably free. Found it necessary to sustain the patient with animal broths, egg, and the farinaceous foods, as she could be induced to take them. Begun at the same time the exhibition of tonics, quinine, tinct. chloride of iron, nux vomica, etc., the latter having a very desirable effect, when the pulse rose, in diminishing its frequency and rendering the pulsations more distinct and vigorous. The case progressed favorably with these measures continued, modified daily to suit the demands of the case, till the end of the third week, when the father of the child, in mistaken zeal for its welfare, (and it being Sunday and he at home all day for the first time), gave it too much of both food and stimulus, bringing on a return of the violent darting pain in the head, raising the pulse from 90 to 130, with great restlessness and distress. Free purgation and renewed blisters, followed by opiates, restored quiet, but could not compensate for the loss in vital condition, the patient being far more exhausted than before. Within a week after the onset of the disease a very troublesome cough set in, not hoarse, and with little expectoration. There being no evidence of either pneumonia or bronchitis I infer that the cough is the result of affection of the origin of the pneumogastric and respiratory nerves. The cough gradually declined as the case has progressed, until it is nearly gone. Subsequently to the relapse the debility is largely increased, and the patient appears as if suffering from a severe typhoid fever, excepting the abdominal symptoms; also has that assemblage of symptoms usually attributed to effusion into the ventricular cavities, which I do not doubt is the case; dilated pupil, diminished sensibility to all outward impressions; slowness of speech and muscular movement; has voided both urine and feces involuntarily; protrudes the tongue when desired, but leaves it between the teeth unless told to retract it; takes food and medicine alike without opposition; occasional sighing. Prof. Gunn saw the case in this condition on Friday last; it was then concluded to devote our whole efforts to sustaining the failing vital power. Ordered beef juice, egg-nogg, Tr. chloride of iron, quinine, nux vomica and oil turpentine, to be given as freely as circumstances would permit. Yesterday had voluntary stool and micturition; would answer when spoken to; subsultus tendinum had ceased, and recognized her parents, and changed her position in the bed. Pushed food, stimulants and tonics. Case terminated fatally May 26th.

CONCLUSIONS FROM THE FOREGOING.

*The onset* is usually sudden; severe symptoms rapidly developed, exciting the gravest apprehension; usually preceded by a chill.

SPECIAL SYMPTOMS.—*Headache* is constant and severe, gradually declining as the case improves. It is sharp, lancinating, the patient often crying out with the severity of the pain.

*Pain in the spine*, like the headache, severe, darting, radiating from the neck to extremities and abdominal and thoracic walls, augmented by muscular movement.

*Cutaneous hyperæsthesia* observed over the whole surface of the body, the patient complaining at the lightest touch, particularly after the case has progressed a few days.

*Spasm* often occurs at the outset, of clonic character. Tonic spasm of spinal muscles, producing true opisthotonos, is a frequent symptom, but probably sometimes simulated by the patient pushing the head back for relief.

*Delirium* is a very constant symptom, early in the disease of active character, and as time goes on, of wandering, muttering, and placid description.

*Stupor* occurs either early in the attack from the overwhelming onset of the disease, and from which the patient arouses into a condition of great pain and distress, or late in disease when there is effusion into the ventricular cavities; or there is general failure of the vital forces and death by asthenia.

*The digestive system* presents in the outset an incontrollable vomiting, frequently of bile, sometimes of mucus only. Constipation is often very great, in fact, almost a constant symptom.

*The urine* in the cases I have observed has constantly been of average amount.

*Respiration* has in all cases been hurried, early in the disease, relatively more than the pulse. Sighing is frequent, and in two cases a troublesome cough appeared to be the result of injury to the respiratory tract at origin of respiratory nerves.

*Cutaneous eruption* is by no means constantly observed. It is petechial, erythematous, or herpetic, but seen only in a minority of the cases, and I do not think has any special significance.

The only item I have to report of morbid anatomy is a number of post mortems by Dr. McKennan, in which he only observed a slight effusion of serum; no pus, organized lymph or effused blood.

Topography.—The cases are scattered about the city, having no relation to special local causes. It is certainly not contagious like variola, measles and typhus, but may be to a certain extent infectious.

Exciting Causes.—It is not the purpose of this paper to speculate upon what may be an exciting cause of this disease, but it is pertinent to mention anything that was probably an exciting cause in either of the foregoing.

Case 1, girl of 10 years, met with a severe blow across the nose from a rope in the hands of her playmates at school. Profuse hæmorrhage, with great pain, followed. The second day after, the disease was developed.

Case 5, girl of 8 years, was bitten by a large dog several weeks before the attack, and terribly frightened, so that she would spring up and cry out in sleep for two or three weeks.

These accidents may have developed the phenomena of the disease, the true cause being latent in the system,—such as is unquestionably the case in yellow fever, and probably in cholera and epidemic dysentery.

Treatment.—As nothing is known of the cause of the disease, prophylactic treatment is wholly out of the question.

If there be a specific poison, (which I do not doubt) we know of no antidote, and hence the treatment of this disease must consist in fulfilling the indications as they appear from time to time.

A great many plans of treatment have been tried,—sedatives, such as aconite, tinct. verat. viride, etc.; also the bromides of potass. and sodæ, the milder anodynes, belladonna, hyoscyamus, cannabis indica, and the like. Tinct. ergot and tinct. calabar bean also, are largely used; all have had their measure of success, and hence all have their advocates. As a consequence, quinine in sedative doses early in the disease, and later in tonic doses, and opiates when there is severe pain, have met with favor also.

Remedial measures may readily be divided into special departments, something as follows:

1st. Efforts to diminish cerebral congestion, general febrile excitement, to allay pain, and procure sleep.

2nd. To maintain special functions in their integrity, as those of the bowels, kidneys and skin.

3rd. To produce a sufficiently profound sedative impression upon the brain and spinal cord to overcome irritability.

4th. To sustain failing vital force by tonics, food and stimulants.

5th. To produce the absorption of effusion into the ventricular spaces, of serum, blood or pus.

The purpose of the first is served by free purging, cold to head and face, warm and stimulating applications to feet, and the exhibition of bromide of potass. or sodium, with tinct. verat. viride or tinct. aconit. rad., and the hot bath. The use of opiates is sometimes appropriate, particularly in those cases where an extraordinary degree of pain of lancinating character is observed. Pulv. ipecac. given with the opium, in equal quantity, enhances its utility, producing a generally harmonious distribution of nerve force, followed by tranquillity and sleep.

There is often found a condition of obstinate constipation, requiring the daily use of some efficient purgative, as the sulphate of magnesia, or, what is better, (except there is some contra-indicating circumstance), a small dose of ol. ricini, with a drop of ol. croton tiglii. The functions of the skin are well maintained by a daily sponge bath with warm water containing an alkaline carbonate in solution, or the addition of a small quantity of cologne spirits or alcohol to the water.

The third, and probably the most important indication, may be met by bromid. potass. and belladonna, tinct. cannabis indica or hyoscyamia, or with tinct. calabar bean, either alone or with ergot. The latter combination has been used with very satisfactory success by many practitioners. The persistent pain in the head so often noticed after convalescence is established, is greatly diminished by a succession of small blisters behind the ears.

It is frequently necessary to early resort to measures to sustain the patient's failing strength, as symptoms ordinarily called *typhoid*, are often rapidly developed; when such is the case no delay should occur, as a single day, or a few hours even, will have the greatest influence upon the result of a case. Beef juice, animal broths, quinine in tonic doses, the finer chalybeates, strychnia, and tinct. cantharidis, are given, adapted to the varying conditions. There is high authority, both American and European, for the use of alcoholic stimulants. As this particular form of inflammation is probably akin to erysipelas as it appears externally, it may be safely concluded that tonics, stimulants and liberal diet must enter largely into the treatment of any case that is at all prolonged, or where the graver symptoms are developed early.

There are frequent recoveries after effusion has taken place, producing its peculiar train of symptoms, including dilatation of the pupil, loss of the sensorial powers, and partial paralysis. I have observed this follow frequent blisters to the back of the neck or behind the ears, the free use of iodide of potassium, with the most active efforts to maintain a normal nutrition of the body.

